# The health sector – led campaign to realise Australia’s national Strategy on health and climate change

**DOI:** 10.1016/j.joclim.2025.100624

**Published:** 2026-03-12

**Authors:** Fiona Armstrong

**Affiliations:** Climate and Health Alliance, 402, Urban Coup, 24 Hope St, Brunswick, Victoria, 3056, Australia

**Keywords:** Climate, Health, Policy, Politics, Australia, Public health, Unfccc, Environment, Advocacy, Campaigns, Community organising

## Abstract

This article outlines some of the key stages in a long-running and groundbreaking campaign for a national strategy on climate and health for Australia. It is hoped this account will be a source of inspiration for others seeking to accelerate climate action in ways that protect and promote health and wellbeing.

A multi-pronged approach combining policy development and advocacy, inside track campaigning, movement building, grassroot engagement, and public communications formed the key elements of the campaign.

## 1 Background

As a major fossil fuel exporter, Australia’s approach to confronting climate change has been deeply political and fragmented, characterised by ‘one step forward -- two steps back’. There has been deep division between the policy positions of the major political parties; climate change agencies have been established and abandoned; and ambition has ebbed and flowed with changes of leadership.

### 1.1 A national alliance of health groups to champion climate action

In August 2010, a group representing around 30 health organisations met with the intention of forming a collective to influence the climate policy discussion in Australia, and the Climate and Health Alliance (CAHA), Australia, was formed. This was the first such national alliance in the world composed of health sector organisations (several others followed, including in Germany, the US, the UK, Ireland, and the US).

Immediately following this inaugural meeting, CAHA called for a national plan on climate change and health. At that time, no health policymakers were paying attention to the issue of climate change, and no climate policymakers appeared to be aware of the significant and deadly impacts for human health in a rapidly warming world.

The Climate and Health Alliance (CAHA) was an initiative championed by the author [1] when turning attention to climate change from health reform in 2010. Eight years working with a coalition of health organisations advocating for health reform (AHCRA) [2], and securing significant reforms of funding and governance of health care [3], made clear the power of advocacy coalitions in achieving significant policy change. The work of the late Australian National University Emeritus Professor Tony McMichael at the intersection of health and climate change served as both inspiration and guidance for this work.

Twenty-eight organisations were willing to join at the outset, and over the following years, many eminent leaders served as leaders and advisors.

It would take 13 years and thousands of conversations to realise the ‘national plan’ imagined in 2010, but in December 2023, a former nursing union colleague, Ged Kearney, now an Assistant Health Minister in the Albanese Labor Government, launched Australia’s first National Health and Climate Strategy at the first ever Health Day at 28th Conference of the Parties (COP28) to the United Nations Framework Convention on Climate Change (UNFCCC), the annual global climate talks in Dubai, United Arab Emirates.

This was a significant milestone, and the result of extraordinary tenacity and commitment by CAHA and its members to persuade a national government (best known for being a laggard on the global climate stage) to embrace the opportunity to realise extraordinary health gains by tackling this interrelationship between the global climate system and the wellbeing of Earth’s dominant -- but now threatened -- species.

### 1.2 The pathway to the national strategy

How can a nation respond to such a complex issue as the health impacts of climate change without a national strategy? This article outlines the work of the Climate and Health Alliance in identifying the need for this policy, then designing a framework to guide policymakers, and advocating for its further development and implementation.

This policy initiative was realised through a multi-pronged approach: combining policy development and advocacy, 'inside track' campaigning, movement building, grassroots engagement, and public communications.

### 1.3 The years 2010-2013: Envisaging key elements and measures of success

#### A yawning gap between evidence and action

At the time of CAHA’s inception, the field of climate change was dominated by physical atmospheric climate and environmental scientists, and impacts on human welfare (societies, health and wellbeing) were not being considered.

Climate change was a novel global health threat, across all aspects of human life, yet no country had developed a plan designed specifically to protect human health from the effects of climate change.

CAHA first called for a national plan on climate and health in 2010; however, it would be three more years before the group (led for the first seven years by a team of volunteers, with limited funding) would scope out the initial key elements of the national plan (later to become the ‘national strategy’).

While significant advances were made on climate policy in the Rudd/Gillard government 2010–2013, scant attention was still being paid to the implications for health by federal, state and territory governments.

Prompted by the opportunity of change offered by the federal election in 2013, CAHA leaders, experts, academics and health professionals worked together to propose an integrated response on climate and health.

Over the next decade, CAHA also contributed to generating evidence for policy, such as contributing to the past six Lancet Countdown Australia Policy Briefs [4].

#### Assessing political parties on climate and health policy

A series of policy priorities formed the basis for CAHA’s first Election Scorecard [5], which assessed six political parties on the basis of their commitments to address the health impacts of climate change and fossil fuels, and to decarbonise healthcare.

The Scorecard was launched along with an online campaigning tool for participants to directly email their Member/s of Parliament and ask them to commit to a National Strategy on Climate, Health and Wellbeing for Australia [6].

Just one party (The Greens) scored more than 1/7 (scoring 6.5 for strong alignment with the CAHA policy asks).

The Coalition of Liberal and National parties, which went on to win the election, each scored 0/7. The Australian Labor Party scored 0.5/7, with just half a point for their commitment to phase out fossil fuel subsidies.

The lack of ambition demonstrated by the Scorecard was only overshadowed by the vigour with which the Coalition government sought to undo the small advances in climate policy made by the previous Labor government, including repealing Australia’s Carbon Price legislation.

What followed the 2013 election was one of the most turbulent periods of national politics in the country’s history. Several Prime Ministerships were won and lost in quick succession over what became known as “The Climate Wars”.

### 1.4 The years 2013–2015: Building power through global networks

Unable to make traction on climate-health policy at the national level, for the next two years (2013–2015), CAHA continued to build sectoral and community support while advocating for fossil fuel phase out on the basis of harmful impacts on human health from coal, and oil, and gas.

#### Going global to influence at home

As a key member of the Global Climate and Health Alliance, CAHA leveraged international pressure to advocate for local policy changes, engaging with global networks to bring about tangible shifts in Australia's climate-health landscape.

Ahead of the important global climate talks in Paris in 2015, CAHA conceived a project and partnered with the World Federations of Public Health Associations (WFPHA) to lead the first-ever global benchmarking survey of national climate and health policy [7].

The intention was to reveal the extent to which countries were (or were not) planning to respond to the unprecedented public health challenge of climate change.

Respondents from 35 countries completed the global survey, which revealed more than half (51 %) did not have an adequate national plan to protect the health of their citizens from climate change. It also revealed Australia lagged behind comparable countries in taking action.

The report from the survey was launched ahead of the United Nations Framework Convention on Climate Change (UNFCCC) negotiations in Paris, supporting the advocacy of international delegates calling for the world’s leaders to prioritise health in the global climate negotiations.

These climate talks would conclude with an important recognition of health in the Paris Agreement, prompting Dr. Maria Neira from WHO to declare: “The Paris Agreement is a Public Health Agreement.”

### 1.5 The years 2016–2018: Engagement and dialogue to build a collective vision

#### Deep engagement laid the foundations

Having clearly demonstrated a huge gap in Australia’s policy landscape, over the next 18 months, CAHA launched a concerted and expansive effort to engage the health sector in responding.

A comprehensive consultation process ensued, involving:

• A Discussion Paper [8] analysing Australia’s climate mitigation and adaptation policies;
• An online survey (Discussion Paper, pp21–28) to gather feedback (July - November 2016);
• An Online Discussion Forum (13–21 August 2016);
• A Health Leaders Roundtable and Meeting with Parliamentarians in Canberra (10 October 2016).

Almost 150 health leaders responded to the online survey [9]; 118 registered and 42 actively participated in the nine day online forum [10]; and forty CEOs, Presidents, and Policy Directors attended a Health Leaders Roundtable Meeting with Parliamentarians in Canberra [11].

The consultation revealed a high level of climate literacy among respondent health professionals, who held serious concerns about the ability of the health workforce to respond effectively to the worsening health impacts of climate change. There was a growing frustration at the failure of the Commonwealth Government to develop effective climate policies or acknowledge and respond to the health risks. And there was a firm view that without a national strategy on climate change and health, Australia would fail to meet its obligations to the health of its citizens under the Paris Agreement.

### 1.6 2017: Surfacing disapproval of government inaction

The national survey found 98 % indicated their support for a national strategy on climate, health and well-being.

The majority of respondents considered the current Federal Government’s climate policies to be ineffective (52 % judged the Coalition’s ‘Direct Action Plan’ to be “not at all effective”).

Significantly, this highly informed group of survey respondents could name almost no policies at either the national or state level that specifically addressed the health impacts of climate change.

#### Despite political division, leaders agree

The Health Leaders Roundtable [11] included representatives from 28 leading medical, public health, nursing, physician, hospital, and community service associations, who sought to express the concerns of their health constituencies and call on politicians to immediately act to respond.

The reception was favourable, with all parliamentarians in attendance acknowledging the importance of the issue and welcoming the leadership from the sector.

Assistant Minister for Health Ken Wyatt expressed his gratitude for this “historic meeting to discuss a climate and health strategy” and said that it was “so important to continue this conversation for our nation” [12].

Shadow Minister for Health Catherine King noted the gap in climate change and health policy, acknowledged other developed nations were doing more, and committed to “work together” with health groups on climate change and health [12].

Greens Leader Dr Richard Di Natale said “the voice of health professionals and organisations was welcome in the climate policy debate” [12].

#### Creating insider consensus on the need for a framework

With strong support for a strategy from the sector and parliamentarians, CAHA set about drafting a Framework for a National Strategy on Climate, Health and Wellbeing for Australia [13].

The Framework outlined policy directions to be taken at the federal, state/territory and local level to:

• protect population health from the impacts of climate change, and
• meet Australia’s international obligations in the Paris Agreement.

This comprehensive policy guidance called on the Federal Government to take a leadership role in the development and implementation of a National Strategy on Climate Health and Well-being, but recognised there was a shared responsibility for implementation across multiple levels, sectors and jurisdictions.

The Framework was launched in the Federal Parliament in June 2017. Two of the major political parties (Greens and Labor) endorsed the framework, and the Assistant Health Minister expressed support.

Shadow Minister for Climate Mark Butler and Shadow Minister for Health Catherine King announced [14] that, when elected to govern, a Labor government would develop a national strategy on climate, health and wellbeing based on the CAHA framework.

So far, so good.

Now it was time to build the movement to create sufficient political pressure to make adoption of the policy politically advantageous (or at the very least, not risky).

The next few years would be spent building power through a community campaign to build pressure on the incumbent Coalition government, as well as hold Labor to account, and to ensure a national climate and health strategy was a key election commitment at the next federal election.

#### Building the movement

The process of engagement with political actors largely occurred using what is called an ‘inside track’ approach. This meant meetings took place via closed doors with decision makers, and hitherto, there was not much of a public-facing campaign. However, given limited engagement with the re-elected Coalition government, political pressure was needed to ensure parliamentarians knew this was a key issue for their constituents, and that they would vote accordingly at the next election.

CAHA focussed on building grassroots power and a political mandate for action by engaging, training and empowering health professionals. A three-day residential [15] program and a series of one-day Climate-Health Champions Training programs [16] taught health professionals about advocacy, campaigning, and building power.

Drawing on the model of community organising used in the wider climate and social justice movement, CAHA worked with allies to develop targeted training, an advocacy toolkit, and campaign posters, tools and resources [17].

Together with face to face and online training, these resources were used to support hundreds of health professionals across the country to develop skills and confidence to front up to their MP, asking them to listen to their concerns and take action to respond.

Over a thousand people emailed their Member of Parliament (MPs) asking them to commit to supporting a national strategy and to take action in the Parliament. CAHA provided coaching for health professionals to meet with their MP and connected people with others locally to join delegations. All MPs were asked to sign a pledge and agree for a photo to be uploaded to the campaign website and shared on social media [18].

A Climate Health Champions Facebook group was established to facilitate ongoing connection and information sharing (now boasting 600 members) as well as key campaign initiatives and events [19].

#### Finding Overton windows^1^

While the conservative Abbott- and Turnbull-led federal governments weren’t interested in climate and health policy advice, state governments were beginning to be: in 2017, the Queensland (Qld) Government were looking for advice on climate and health policy to guide the development of an adaptation plan for the state. Finding none (apart from CAHA’s framework), the Queensland Department of Environmental and Science (DES) invited CAHA to work with the National Climate Change Adaptation Research Facility (NCCARF) to write the first climate and health adaptation plan for Queensland [20].

The Human Health and Wellbeing Adaptation Plan for Queensland (2018) was the first state-wide climate and health plan produced in Australia, and it drew heavily on CAHA’s framework. The consultation that informed the Queensland plan revealed less than 12 % of respondent hospitals and health services had conducted a climate risk assessment on their operations, facilities, workforces or communities, prompting the development of a climate risk strategy [21] and climate risk management tools [22] for hospitals and health services by the Queensland Department of Health, the first jurisdiction in Australia to do so.

#### 1.6.1 2019: Harnessing dramatic climatic events to build political will

The summer that preceded the federal election in May 2019 was epic in the scale of environmental and climate catastrophes: over a million fish died when parts of the nation’s largest inland river system dried up [23]; half a million cattle were lost in floodwaters in the north, [24] and the cooler southern island of Tasmania experienced raging bushfires [25]. Despite the absence of any credible climate policy (and 29 % of the voters rating the environment as their biggest concern) [26], former Treasurer Scott Morrison was elected Prime Minister.

Later that year, Australia would be accused at the global climate talks of obstructing progress of the Paris climate agreement, and would experience bushfires so intense that the smoke circumnavigated the globe. Over a billion animals [27] and 445 people died, over 4000 people went to hospital [28], and 19 million hectares of land burned [29], in what became known as ‘Black Summer’ -- an event during which the Prime Minister would secretly holiday in Hawaii, rejecting calls for him to return by famously telling the media: “I don’t hold a hose, mate.” [30]

#### Local governments seek guidance

The health impacts of climate change were becoming very real for many communities around the country, including in one of Australia’s most diverse and populated urban areas: western Sydney. In 2019, stakeholders from the Western Sydney Health Alliance approached CAHA to ask it to lead the development of climate and health policy guidance for local government stakeholders in one of Australia’s most climate vulnerable regions.

The Climate, Health and Wellbeing Framework produced for The Parklands Councils in Western Sydney was based on the eight Areas of Policy Action in CAHA’s Framework, and added one more: Collaborative Partnerships for Action [31].

CAHA’s guidance for Western Parkland City was the first of its kind in Australia and served to support local government stakeholders to embed climate and health in local government decision making, strategic planning, policy development and service delivery.

### 1.7 The years 2020–2023: Building capacity and interest at state government level

#### Building commitment and connection among state/territory governments

During the period of the Coalition government (2010–2022), CAHA engaged frequently with state and territory governments and international stakeholders. Together with state-based actors, CAHA led advocacy efforts calling on state and territory governments to prioritise climate and health.

CAHA lobbied the Australian Capital Territory Government’s (ACT) Climate Change and Health Ministers over several years to recognise and respond to the health impacts of climate change. The ACT government has now committed to achieve net zero emissions for the public health sector by 2040, and has established a healthcare decarbonisation roadmap. In 2020, the ACT Government announced the territory’s [32] first all electric hospital, allowing the hospital to become one of the first entirely powered by renewable energy.

In Western Australia (WA), CAHA worked alongside state-based groups to champion the inclusion of environmental sustainability in the state’s Sustainable Health Review at a Climate and Sustainability Forum [33]. CAHA participated as an expert witness at the subsequent WA Climate and Health Inquiry, which responded to collective advocacy and led to the establishment of a Sustainable Development Unit in the Department of Health [34].

Together with Queensland stakeholders, CAHA called for the implementation of Priority Adaptation Measures it had outlined in the HCAP report [35]. The subsequent establishment of an Office of Hospital Sustainability and a $30 million solar panel and energy efficiency program to install solar generation (solar panels) at 50 hospital sites were among the most significant measures adopted.

In 2020, CAHA attended a Roundtable on Climate Change and Health at the invitation of the Tasmania Government’s Department of Health, with the subsequent policy report adopting the Areas of Policy Action outlined in CAHA’s Framework and identifying actions that could be taken in Tasmania [36].

In 2022, the New South Wales (NSW) Ministry of Health joined several other state and territory governments in joining CAHA’s Global Green & Healthy Hospitals Pacific network (GGHH Pacific) as a member and announced the establishment of a new Climate Risk & Net Zero Unit to scale up and coordinate environmental sustainability actions across the public health system in NSW.

#### Using futures thinking

Then, of course, in early 2020, came COVID. As in many times in the past, the team at CAHA shifted tack. This was an opportunity from the disruption of COVID-19 as a period of reflection -- and imagining a better future.

Inspired by an article in the Journal of Futures Studies by UNESCO Chair in Futures Studies Sohail Inayatullah and OneHealth Foresight Consultant Peter Black [37] on whether COVID-19 was a ‘black swan event’ (i.e., totally unpredictable -- spoiler, it wasn’t), CAHA sought Professor Inayatullah’s advice in designing a futures thinking exercise on climate change and health.

Over the period of six months in 2020, CAHA recruited over 100 transdisciplinary thought leaders from Australia and around the world to engage in ‘Rewriting the Future’ [38].

With the support of another futures leader, Dr. Colin Russo, over three roundtables over a six week period, this group brainstormed several possible alternative futures, and built detailed scenarios and case studies for each of them. The preferred future, Our Island Home, imagines a future in which both societal and planetary health are thriving.

#### Healthy, regenerative and just – the world we want

The report from this exercise, “Australia in 2030: Possible Alternative Futures" [39], described five scenarios based on different combinations of social, technological, environmental, ethical, governance, legal, and economic factors.

Four narratives sought to answer the questions: "What will Australia look like if over the next decade there is: no change; marginal change; maladaptive change; or radical transformative change?"

A fifth integrated scenario was developed using a process of 'backcasting,' i.e., how do we get to our preferred future?

This informed the development of the CAHA Framework 2.0: Healthy, regenerative and just Ppolicy agenda -- The roadmap to our preferred future [40].

This contained 175 policy recommendations across eight critical areas of policy action, spanning multiple portfolios, and outlining actions for federal, state and local governments, business, community, and the health sector.

### 1.8 2021-2024: Leveraging connections with global actors

Ahead of COP26, CAHA partnered with World Health Organization and the UK Foreign Commonwealth and Development Office (representing the COP Presidency) to host a Climate Health Leadership Roundtable with state and territory health ministers and officials to promote the opportunities available in the Alliance for Transformative Action on Climate and Health (ATACH) from WHO [41]. This provided an opportunity for Australian state and territory health actors to meet with global counterparts to learn about climate action for health across multiple jurisdictions and countries.

#### Finally, the ‘climate election’

With a mix of inside track and public campaigning, CAHA contributed to climate change being a key election issue in 2022 [42].

As in earlier years, CAHA produced a scorecard on the performance of political parties against 17 key climate and health policies [43]. The scorecard was distributed to all Members of Parliament (MPs), CAHA’s networks, and to Australian media -- conveying the important message that the health community cared about climate action -- and many were likely to vote accordingly.

Working with allies in the environment movement, CAHA also worked to target voters in 20 electorates around the country where there was a strong sentiment in favour of climate action. CAHA led in four key electorates where there was an active network of climate-concerned health professionals, climate and health policy commitments by candidates, and voters who indicated climate and health were key priorities [42].

The election results were dramatic: according to official polling, climate change was the most important issue for voters. The Australian Greens gained a record number of seats, nine independent candidates with strong climate action platforms were elected (many in disaffected historically conservative electorates) and the Australian Labour Party, having endorsed *Healthy, Regenerative and Just*, and committed to a suite of climate policies, was elected to govern.

In each of the four electorates where CAHA and allies elevated climate-concerned health voices, a more climate-friendly party was elected [42].

#### The development of the national strategy

Following the election, AUS $3.4 million was allocated to establish a unit in the Commonwealth (federal) Department of Health to lead on the development of the Strategy: the National Health, Sustainability and Climate Unit [44]. In October 2022, CAHA was invited, along with around 15 other stakeholders, to join a committee advising the office of the Chief Medical Officer on the development of the strategy. A stakeholder consultation period followed, with 16 workshops held across the country. A thematic report published in October 2023 [45].

The imminent first ever Health Day at COP28 provided a short deadline for the Strategy’s development, with the federal government keen to regain credibility on climate by using this opportunity to launch the Strategy.

Assistant Minister Ged Kearney launched the Strategy on 3rd December, and announced Australia would join the global Alliance for Transformative Action on Climate and Health (ATACH) [46].

The National Health and Climate Strategy committed to action over five years with a vision for ‘Healthy, climate-resilient communities, and a sustainable, resilient, high-quality, net zero health system’.

The Strategy drew on many elements and recommendations from CAHA’s Framework. For example, the Strategy similarly contained:

• four key objectives to:
  ○ build a climate resilient health system and capacity to protect population health (reflected recommendations in CAHA’s Framework Area of Policy Action #2);
  ○ build a sustainable, high quality net zero health system (aligned with Area of Policy Action #6);
  ○ collaborate internationally (consistent with Area of Policy Action #8); and
  ○ support healthy climate resilent and sustainable communities (consistent with Area of Policy Action #1, 2, 4, 5 & 7)
• and four enablers of action:
  ○ workforce, leadership, and training (echoing Area of Policy Action #5, 6 & 8);
  ○ research and innovation (drawing on Area of Policy Action #7);
  ○ communication and engagement (consistent with Area of Policy Action #5); and
  ○ collaboration and governance (aligned with Area of Policy Action #8)

The Strategy itself was launched by Minister Kearney at COP28 [47], highlighting the following key elements:

• a national health vulnerability capacity and adaptation assessment;
• a plan to decarbonise the Australian health system, produce a baseline emissions estimates for the health system;
• sustainability and climate resilience standards for healthcare;
• identification of emissions hotspots from buildings, transport and the healthcare supply chain;
• identification of opportunities for knowledge sharing; and
• collaboration with other jurisdictions to align procurement requirements and footprinting standards.

### 1.9 The state of play in 2025

By developing Australia’s first National Health and Climate Strategy, the Australian Government committed to making climate change a national health priority.

At time of writing, however, insufficient funding has been committed to the implementation of the Strategy [48]. Despite this, progress is being made by the dedicated team in the Health Sustainability and Climate Unit (established in 2023) in the Commonwealth Department of Health, with support from the interim Centre for Disease Control, with an Implementation Plan developed in late 2024.

An update on progress released in early 2025 reveals: baseline estimates of health sector emissions have been developed; work has begun on new energy efficiency ratings tools for private hospitals and medical centres; progress is being made towards the phase out of emissions-intensive desflurane; and national health system guidelines are being developed for green procurement and sustainable resource use [49].

The Commonwealth is also collaborating with others, having joined a consortium convened by Monash Sustainable Development Institute to develop a guide to healthcare decarbonisation [50].

There has also been some further progress at state level, with South Australia Health launching its own Climate Change and Health framework and New South Wales launching a Net Zero Roadmap for the state’s health system, both in June 2025.

However, while there are many willing stakeholders (including sub-national governments), much more is needed from the federal government to see the National Health and Climate Strategy fully implemented. So, “it ain’t over, till it’s over”, as Lenny Kravitz would say.

**Table:** 

How a campaign built a movement While the progress towards climate and health policy in Australia through this campaign were important, the activities, engagement, capacity building and collective action contributed to something even more powerful and enduring: a strong and influential climate and health movement. During the period of this campaign, CAHA’s membership grew from less than 30 to over 100 organisational members representing 70 different health professions, its social media channels grew, the CAHA team became 13, and annual income grew from less than $10,000 to $1,000,000. CAHA’s email list of supporters increased to 12,000, and social media channels grew to 9,000 on X/Twitter, 6,000 on Facebook, 4,000 on LinkedIn, and 1,500 on Instagram [51].

## 2 Conclusion

A combination of leadership in policy innovation and policy advocacy, movement building, communications and grassroots campaigning contributed to the achievement of Australia’s national health and climate strategy. Against a background of a fraught political landscape, perseverance in garnering support across Australia's health sector finally won the day. The leadership of CAHA in defining and describing the need for, and the elements of, the national strategy was the key to the issue getting on the radar of politicians and persuading them there was strong support in the sector (and more broadly) for a coordinated approach to climate and health policy.

There is still a ways to go in realising the full implementation of the strategy. However, the pathway to its development, as well as the goals and objectives it sets out, offers important lessons for advocates, policymakers and concerned citizens about the nature of policy making and the kinds of actions that can bring complex policy to life.

## Author agreement

The author hereby grants to the *Journal of Climate Change and Health* the exclusive right to publish the manuscript. The author agrees to credit the journal as the original place of publication. The author grants the journal the right to the manuscript, the copyright, and the revisions. The Journal has the right to reproduce, publish, and distribute copies of the manuscript. It has the right to re-print the manuscript. Upon the acceptance, the author’s name will always be included with the publication of the manuscript.

## CRediT authorship contribution statement

**Fiona Armstrong:** Writing – original draft, Project administration, Conceptualization.

## Declaration of competing interest

The authors declare that they have no known competing financial interests or personal relationships that could have appeared to influence the work reported in this paper.

## Footnotes

1 Named after the American policy analyst Joseph Overton, an Overton window refers to the range of policies a politician can recommend to keep public office given public opinion at that time.
